# AI-driven warehouse automation: a unified edge computer vision framework for real-time package tracking and conveyor monitoring

**DOI:** 10.3389/frai.2026.1917595

**Published:** 2026-10-29

**Authors:** Mohammed Mahyoub, Ankit Pandey, Farouq Halawa, Raashid Mohammed

**Affiliations:** Science and Advanced Concepts, NA Ops Engineering, Amazon, Bellevue, WA, United States

**Keywords:** computer vision, conveyor monitoring, edge deployment, real-time object tracking, trajectory analysis, virtual sensors, warehouse automation

## Abstract

High-speed warehouse automation depends on reliable monitoring of package flow and operational anomalies across sortation conveyors, yet conventional solutions typically deploy separate physical sensors for individual tasks. This paper presents a unified edge computer vision framework that derives multiple conveyor-monitoring functions from a single overhead camera and a shared detection-tracking pipeline. The key novelty is the use of continuous, identity-preserving package trajectory tracking and analysis as a shared basis for *virtual sensors*: software-defined trigger lines and detection zones. Throughput counting, doubles detection, and reversal detection are derived from the shared trajectories, while dwelling detection reuses the same per-frame detections for a periodic zone-occupancy check; all four functions are therefore served by one detector pass per processed frame, without task-specific sensing hardware or redundant inference pipelines. The framework combines a fine-tuned RF-DETR Small detector with ByteTrack multi-object tracking to generate identity-preserving package trajectories in real time. It was evaluated at an operational sortation facility, where throughput counting achieved 98.05% accuracy; doubles detection achieved 96.0% precision and reversal detection 96.6% precision, while event recall, estimated on approximately six hours of continuously annotated video, was 66.7% for doubles and 88.9% for reversals; and dwelling detection achieved 100% precision and 96.15% recall. These results show that a shared detection and trajectory-analysis backbone can provide high-precision, reconfigurable multi-task conveyor monitoring on edge hardware while supplying trajectory and video metadata unavailable from binary physical sensors, offering a practical building block for AI-driven warehouse automation.

## 1 Introduction

The sustained growth of e-commerce logistics has driven the adoption of high-speed sortation systems (Jain et al., 2021). Within these facilities, induction points serve as the critical interface where packages transition from upstream processing onto the sortation conveyor for outbound delivery. Maintaining optimal throughput at each induction point requires continuous monitoring for operational anomalies. These include miscounted packages, simultaneous multi-package inductions (doubles), directional reversals caused by mechanical or sensing faults, and packages that stall on the conveyor surface.

Traditionally, each monitoring objective has been addressed through dedicated physical sensor arrays: photoeyes, break-beam counters, and proximity sensors (Takawade et al., 2024; Sinaga et al., 2025; Aung et al., 2019). These devices are expensive to install, prone to signal jittering, and limited to binary event detection without contextual metadata or visual evidence. A single physical reversal, for example, can trigger multiple photoeye activations in rapid succession, making accurate event counting unreliable.

Recent studies demonstrate the value of artificial intelligence for industrial inspection and process monitoring. Ahmad et al. evaluate EfficientDet backbones for defect detection on fast industrial conveyors and explicitly analyze the trade-off among detection accuracy, computational cost, and conveyor timing (Ahmad et al., 2026). Such work establishes the importance of reliable perception and computationally feasible deployment in industrial environments. However, it focuses on defect detection as an individual function and does not use continuous, identity-preserving package trajectories to derive multiple operational monitoring outputs from a shared vision pipeline.

Recent advances in real-time object detection (Robinson et al., 2025; Zhao et al., 2024) and multi-object tracking (Zhang et al., 2022) create an opportunity to complement, and for selected monitoring functions potentially substitute for, separate sensor arrays with a single overhead camera. A camera positioned above a conveyor segment can observe all packages traversing the field of view and produce continuous trajectory data, from which diverse operational metrics can be extracted simultaneously. Existing work in warehouse automation and logistics inspection (Halawa et al., 2025; El-sayed et al., 2022) has largely focused on individual tasks in isolation, such as package counting, defect detection, or jam identification. None of these efforts demonstrate how continuous tracking and trajectory analysis can serve multiple monitoring objectives concurrently in a production setting.

The objective of this study is to determine whether continuous, identity-preserving package trajectories obtained from a single overhead camera can serve as a shared basis for multiple conveyor-monitoring functions on resource-constrained edge hardware, as a potential alternative or complement to the task-specific physical sensors traditionally deployed at each induction point.

The primary contributions of this work are:

- A unified, edge-deployable perception architecture that couples RF-DETR Small detection with ByteTrack to construct continuous, identity-preserving package trajectories from a single overhead camera stream. The architecture makes one shared detection output and trajectory representation available to multiple downstream monitoring functions, avoiding separate detection and tracking pipelines for each function.
- A virtual-sensor formulation that transforms tracked package trajectories into operational events through lightweight spatial and kinematic computations. The formulation instantiates three trajectory-based functions (trigger-line crossing for throughput counting, a two-line temporal state machine for doubles detection, and displacement and velocity alignment for reversal detection) and one detection-based function, periodic zone occupancy for dwelling detection, which reuses the shared detections without tracking state.
- An operational evaluation at a sortation facility that validates the shared detection and trajectory-analysis approach across four monitoring functions: 98.05% throughput-counting accuracy, 96.0% doubles-detection precision (66.7% recall), 96.6% reversal-detection precision (88.9% recall), and 100% dwelling-detection precision with 96.15% recall. The evaluation demonstrates that the proposed architecture can support high-precision, reconfigurable multi-task monitoring on edge hardware, and quantifies the remaining false-negative rate for doubles and reversal detection.

To meet this objective, we develop a framework built on two core capabilities: real-time package tracking and trajectory analysis. A fine-tuned RF-DETR detector combined with ByteTrack provides continuous, identity-preserving localization of every package in the camera field of view, and trajectory analysis over these tracked positions derives three operational outputs: throughput counting, doubles detection, and reversal detection. A fourth output, dwelling detection, applies a periodic zone-occupancy test to the same shared detections. The abstraction enabling this approach is the *virtual sensor*: a software-defined trigger line or detection zone evaluated geometrically over the shared trajectories or detections, so that each monitoring capability is added without additional hardware or redundant inference passes.

The remainder of this paper is organized as follows. Section 2 reviews related work in object detection, multi-object tracking, and warehouse automation. Section 3 details the methodology, including the detection and tracking pipeline, the virtual-sensor formulation, the four monitoring algorithms, and the edge deployment architecture. Section 4 presents the operational evaluation results. Section 5 discusses computational efficiency, virtual-versus-physical sensing, deployment lessons, transferability, and limitations. Section 6 concludes.

## 2 Related work

### 2.1 Object detection

Object detection is a fundamental task in computer vision that involves localizing and classifying objects within an image. The field has evolved significantly since R-CNN (Girshick et al., 2014), which introduced a multi-stage process that refines candidate regions through CNN feature extraction before classifying them with an SVM. While pioneering, this approach proved computationally intensive for real-time inference. Subsequent work improved efficiency through Region of Interest (RoI) pooling and the Region Proposal Network (RPN) (Girshick, 2015; Ren et al., 2016).

Real-time inference remained a primary challenge until the You Only Look Once (YOLO) family of single-stage architectures emerged. These models process an entire image in a single pass to predict bounding boxes and class probabilities simultaneously, achieving processing rates as low as 22 milliseconds per image (Redmon et al., 2016; Redmon and Farhadi, 2018). While YOLO prioritized speed, its accuracy on benchmark datasets such as Microsoft COCO (Lin et al., 2014) was initially limited. The research community has since produced numerous iterations, including YOLOv4, YOLOv7, and more recent versions such as YOLOv8 and YOLOv11 (Bochkovskiy et al., 2020; Wang et al., 2023; Varghese and Sambath, 2024; Khanam and Hussain, 2024).

Further developments led to high-accuracy, single-stage models like RTMDet, which optimizes the balance between speed and precision (Lyu et al., 2022). However, most of these models rely on convolutional neural networks (CNNs), which excel at capturing local features but struggle with long-range dependencies within an image. Transformer-based detection models address this limitation through self-attention mechanisms, offering enhanced accuracy at the cost of inference speed (Carion et al., 2020; Zhao et al., 2024). More recently, the RF-DETR family leverages recurrent features and transformer architectures to achieve both fast inference and high detection accuracy, making it highly suitable for high-speed industrial applications (Robinson et al., 2025).

### 2.2 Multi-object tracking

In dynamic environments where object locations change over time, such as vehicles on roadways, pedestrians in urban spaces, or packages on conveyor systems, consistent temporal tracking is crucial for real-time monitoring. Several tracking algorithms address the challenges of occlusion and detection variance. ByteTrack introduces the BYTE association method, which leverages all detection boxes rather than only high-score ones. By associating low-score detection boxes with existing tracklets based on their similarities, ByteTrack effectively recovers objects that might otherwise be missed due to motion blur or partial occlusion (Zhang et al., 2022).

Other prominent algorithms, such as BoT-SORT, combine motion and appearance information with camera motion compensation. This approach fuses the Intersection over Union (IoU) distance matrix with a Re-identification (ReID) cosine distance matrix to achieve more robust associations between detections and tracklets (Aharon et al., 2022). To handle non-linear motion, OC-SORT updates object track histories based on observations from the detection model to maintain consistency during trajectory interruptions (Cao et al., 2023). A variant of this, Deep OC-SORT, further integrates adaptive appearance matching to enhance tracking reliability (Maggiolino et al., 2023). While newer architectures continue to iterate on these foundations (Yang et al., 2024; Cao et al., 2025; Yi et al., 2024; Chao et al., 2025; Huang et al., 2024), ByteTrack remains a highly robust choice for industrial conveyance systems, where objects typically follow linear trajectories and high-speed processing is required.

### 2.3 Warehouse automation and logistics inspection

Logistics inspection has evolved from manual methods to autonomous systems that leverage the detection and tracking architectures discussed above. Early work emphasized intelligent perception for robust material handling, utilizing modular frameworks for object detection and grasp planning in dynamic industrial environments (Poss et al., 2020). As logistics complexity increased, research shifted toward structural recognition, employing CNN-based instance segmentation to verify the arrangement of multiple transport units with high precision (Dörr et al., 2020).

For real-time tracking on conveyor systems, deep learning significantly outperforms conventional image processing. El-Sayed et al. demonstrated that YOLOv5 achieves nearly 98% accuracy at frame rates suitable for industrial throughput (El-sayed et al., 2022). More recently, Halawa et al. (2025) introduced an IIoT framework for real-time conveyor monitoring that benchmarks state-of-the-art detectors, including YOLO variants, RTMDet, and RT-DETR, for identifying diverse package types, and demonstrates novel conveyor extraction algorithms for jam detection and throughput monitoring in operational settings. Recent advancements have moved toward specialized architectures, such as two-dimensional flow models that integrate multi-scale feature fusion and probabilistic transformations to maintain robustness under high variability and complex logistics conditions (Huang, 2025). Collectively, these developments, often framed within the “Packaging 4.0” paradigm, highlight how AI and machine learning have become transformative tools for optimizing quality inspection, fraud detection, and overall supply chain efficiency (Kumar et al., 2025).

Adjacent industrial studies further demonstrate the breadth of AI-enabled monitoring. Ahmad et al. (2026) optimize backbone selection and conveyor timing for real-time visual defect detection. Such approaches address inspection as a distinct task. In contrast, the present work uses continuous package trajectories to derive several conveyor-monitoring outputs from one shared perception pipeline.

### 2.4 Comparative analysis and research motivation

Table 1 compares representative prior studies using the capabilities most relevant to multi-task conveyor monitoring. A check mark indicates that the capability is explicitly addressed or evaluated in the cited work; a dash indicates that it is not reported as part of that study.

**Table 1:** Capability comparison of representative related work and the proposed framework.

Study	Vision	ID track.	Multi-task	Real-time	Shared traj.
Ahmad et al. (2026)	✓	–	–	✓	–
El-sayed et al. (2022)	✓	✓	–	✓	–
Halawa et al. (2025)	✓	✓	✓	✓	–
Ning et al. (2022)	–	–	–	–	–
**This work**	✓	✓	✓	✓	✓

The comparison shows that prior work has established effective solutions for visual inspection, process-quality prediction, package tracking, and conveyor monitoring. However, these capabilities are generally addressed independently. The proposed framework combines vision-based perception, persistent package identification, and trajectory-derived event analysis in a shared edge-deployable pipeline. This shared representation supports four monitoring outputs without separate detection and tracking pipelines for each function: throughput counting, doubles detection, and reversal detection are derived from the shared trajectories, and dwelling detection from the shared detections.

### 2.5 Research gaps and industrial deployment challenges

Despite significant advancements in object detection, multi-object tracking, and automated inspection, a critical gap remains in deriving multiple operational metrics from a single shared vision pipeline on high-speed logistics conveyors. Existing approaches typically address individual monitoring tasks in isolation: throughput counting via photoeye sensors, anomaly detection via dedicated hardware triggers, or quality inspection via offline image analysis. Each requires per-task sensing infrastructure that scales poorly across many monitoring points. For instance, Ning et al. (2022) utilize RFID-based revolving scanning to detect package structures, while conventional reversal detection relies on mechanical photoeye arrays that are prone to signal jittering and cannot distinguish between distinct physical events. These sensor-based methods do not generalize across tasks and offer no trajectory-level understanding of package behavior.

Furthermore, there is a notable scarcity of end-to-end computer vision pipelines that move beyond laboratory benchmarks to successful deployment in high-throughput industrial environments. Most existing studies prioritize algorithmic accuracy on curated datasets over the computational efficiency, memory management, and system stability required for continuous 24/7 operations on resource-constrained edge hardware. This research addresses these combined gaps by presenting a unified tracking and trajectory analysis framework in which a single detection and tracking pass produces shared detections and per-package trajectories, from which multiple monitoring capabilities are derived as lightweight geometric computations: throughput counting, doubles detection, and reversal detection from the trajectories, and dwelling detection from the detections. The system is deployed on edge hardware, demonstrating that real-time multi-task conveyor monitoring is achievable without redundant inference passes or dedicated per-task sensors.

## 3 Methodology

This section presents the computer vision pipeline developed for real-time package tracking and trajectory analysis on high-speed conveyor systems. The methodology is organized as follows: Section 3.1 provides a system overview and introduces the virtual sensor abstraction; Section 3.2 establishes the shared mathematical notation for virtual sensors; Section 3.3 describes the package detection model; Section 3.4 details the multi-object tracking approach that builds per-package trajectories; Section 3.5 formalizes throughput counting derived from trigger-line crossings; Section 3.6 presents the doubles detection state machine; Section 3.7 defines the trajectory-based reversal detection algorithm; Section 3.8 describes the dwelling detection procedure; Section 3.9 discusses the edge deployment architecture; and Section 3.10 describes how the algorithm parameters were selected. The complete tracking pipeline is formalized in Algorithm 1.

Algorithm 1 Real-time package tracking and trajectory analysis. 
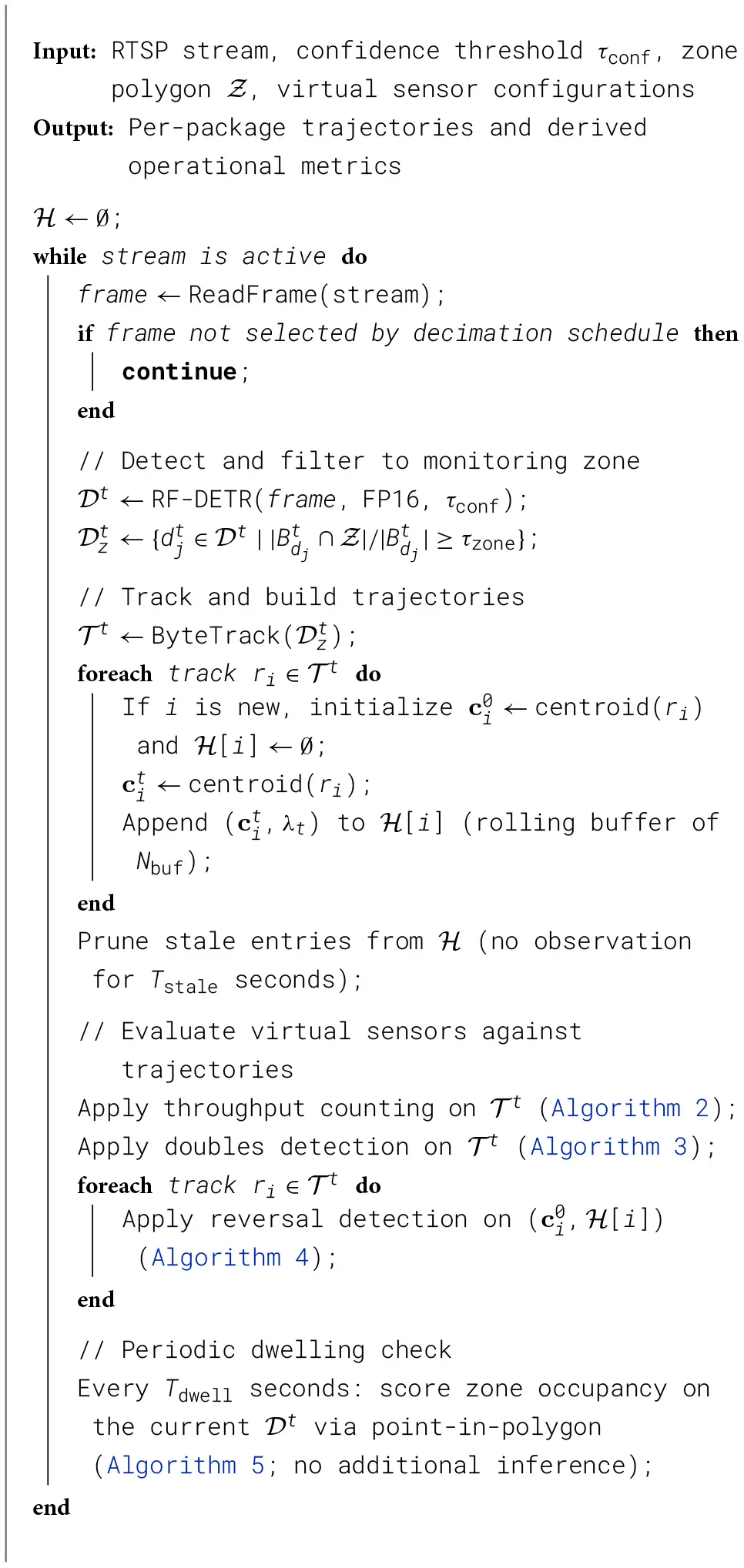

### 3.1 System overview

The proposed system continuously tracks packages as they traverse the camera field of view and builds per-package trajectory histories from which multiple operational metrics are derived. A single overhead camera is installed at each induction point, capturing a live video stream via the Real-Time Streaming Protocol (RTSP) at 1,920 × 1,080 resolution and 30 frames per second (FPS). The core processing loop performs object detection on each selected frame, assigns persistent identities to detected packages through multi-object tracking, and appends each new centroid observation to a per-track trajectory buffer. These trajectory buffers constitute the central data structure of the system: every downstream monitoring output is computed by evaluating geometric or kinematic conditions against the accumulated trajectory data.

Rather than deploying separate physical sensors for each monitoring objective, the system introduces the concept of *virtual sensors*: software-defined spatial primitives (trigger lines and detection zones) that are evaluated against package observations to produce task-specific outputs. Four monitoring capabilities are derived without additional hardware or redundant inference passes: (1) throughput counting, which registers packages as their trajectories cross a virtual trigger line; (2) doubles detection, which applies a two-line crossing state machine to identify simultaneously inducted packages; (3) reversal detection, which analyzes trajectory displacement and velocity vectors to identify packages that reverse direction; and (4) dwelling detection, which periodically scores the detection zone for stationary packages. Throughput counting, doubles detection, and reversal detection operate on the shared trajectories produced by a single detection and tracking pass; dwelling detection does not use the trajectories and instead applies a stateless zone-occupancy test to the detections already produced by the same pass at its scoring interval, without additional inference. The computational cost of adding each monitoring output is therefore limited to lightweight geometric or vector computations. Figure 1 illustrates the physical layout of a single induction point and the spatial arrangement of virtual sensors.

**Figure 1:**
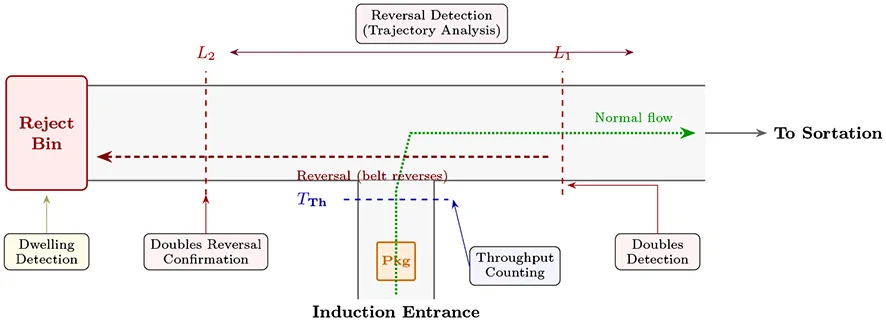
Induction point layout showing the conveyor path from entrance to reject bin. Three virtual trigger lines are configured: *T*_Th_ for throughput counting, *L*_1_ for doubles first-line detection, and *L*_2_ for doubles reversal confirmation. Packages normally flow from the entrance through *T*_Th_ and right toward sortation; reversed packages travel back past *L*_2_ into the reject bin, where dwelling detection monitors for stationary packages.

The system employs a multi-threaded architecture to decouple input/output operations from GPU-bound inference. Five concurrent threads operate in a producer-consumer pattern: (1) a frame capture thread that reads from the RTSP stream and enqueues raw frames; (2) a processing thread that performs detection, tracking, trajectory maintenance, and virtual sensor evaluation on each frame; (3) a video save thread that encodes recorded event clips to disk; (4) an upload thread that transmits video files and metadata to cloud storage; and (5) a log management thread that periodically uploads diagnostic logs. This design ensures that network latency during cloud uploads does not impede real-time frame processing, and that disk I/O during video encoding does not introduce inference delays.

### 3.2 Mathematical formulation of virtual sensors

At processed frame *t*, let $D^{t}=\left\{d_{j}^{t}\right\}$ denote the raw detector outputs, indexed by *j*. After association, let $B_{i}^{t}=\left(x_{i,\min}^{t},y_{i,\min}^{t},x_{i,\max}^{t},y_{i,\max}^{t}\right)$ denote the bounding box assigned to persistent track *i*. The corresponding package centroid is


$$\begin{array}{l} {\text{c}}_{i}^{t}=\left(\frac{x_{i,\min}^{t}+x_{i,\max}^{t}}{2},\frac{y_{i,\min}^{t}+y_{i,\max}^{t}}{2}\right)\in {\mathbb{R}}^{2}. \end{array}$$ (1)

For each track identity, the system maintains a timestamped trajectory buffer


$$\begin{array}{l} H_{i}^{t}=\left\{\left({\text{c}}_{i}^{s},{\text{λ}}_{s}\right)|\max\left(0,t-N_{\text{buf}}+1\right)\leq s\leq t\right\}, \end{array}$$ (2)

where λ_*s*_ is the timestamp of processed frame *s* and *N*_buf_ is the maximum buffer length. This trajectory representation is shared by all virtual-sensor modules.

A virtual trigger line *L* is represented by an anchor point ${\text{p}}_{L}\in {\mathbb{R}}^{2}$ and a unit normal vector **n**_*L*_. The signed distance of a centroid from the line is


$$\begin{array}{l} g_{L}\left(\text{c}\right)={\text{n}}_{L}^{⊤}\left(\text{c}-{\text{p}}_{L}\right). \end{array}$$ (3)

For the direction σ ∈ {−1, +1}, a directed crossing of *L* is defined by


$$\begin{array}{l} \chi_{L,\sigma }^{t}\left(i\right)=\text{𝕀}\left[\sigma g_{L}\left({\text{c}}_{i}^{t-1}\right)<0\wedge \sigma g_{L}\left({\text{c}}_{i}^{t}\right)\geq 0\right], \end{array}$$ (4)

where *𝕀*[·] is the indicator function. This predicate provides the common geometric basis for throughput counting and the two-line doubles state machine.

Similarly, a virtual detection zone Ƶ is represented as a polygon, with centroid membership defined as


$${\text{𝕀}}_{Ƶ}(c)=\{\begin{array}{ll} 1, & c\in Ƶ,\\ 0, & c\notin Ƶ. \end{array}$$ (5)

The trajectory-based monitoring outputs are task-specific functions of the shared trajectory and the corresponding virtual-sensor configuration:


$$\begin{array}{l} e_{m}^{t}\left(i\right)={\text{Φ}}_{m}\left(H_{i}^{t},G_{m};\theta_{m}\right),\;m\in \left\{\text{throughput},\text{doubles},\text{reversal}\right\}, \end{array}$$ (6)

where $G_{m}$ contains the virtual lines and zones required by task *m*, and **θ**_*m*_ denotes its thresholds and timing parameters. Dwelling detection does not use the trajectory representation; it is stateless and instead applies the zone predicate in Equation 5 directly to the detections already produced by the shared detector for the current processed frame at each scoring interval. The following subsections specify each monitoring function and its associated algorithm.

### 3.3 Package detection model

Reliable detection forms the perception layer upon which all downstream tracking and trajectory analysis depends. The detection backbone is RF-DETR Small (Robinson et al., 2025), a transformer-based object detection architecture that combines recurrent feature refinement with the Detection Transformer (DETR) paradigm. RF-DETR was selected for its favorable balance of detection accuracy and inference speed on edge hardware, achieving state-of-the-art performance on the COCO benchmark while maintaining real-time throughput. The model was fine-tuned on a proprietary dataset of 1,920 × 1,080 images collected from overhead cameras installed on warehouse conveyance systems. To diversify the dataset, images were captured across multiple sites and multiple camera views, and frames were sampled at widely spaced time intervals to avoid including near-duplicate snippets of the same scene. The resulting images were then partitioned randomly at the image level into training, validation, and test sets following an 80/10/10 ratio. Because the split was performed at the image level rather than by camera, site, or recording session, images from the same cameras and sites, including the facility used for the operational evaluation, appear in all three subsets. Temporal spacing of the sampled frames reduces near-duplicate content, but it does not remove the similarities shared by images from a common camera, namely the fixed conveyor background, camera geometry, and lighting. The held-out test set therefore measures detection performance under acquisition conditions related to those seen in training, rather than generalization to unseen cameras or sites. This dataset comprises 3,400 training images containing 8,232 annotated instances, 425 validation images with 2,544 instances, and 425 test images with 2,933 instances. The fine-tuned model recognizes two object classes: boxes (class ID 0) and envelopes (class ID 1), which together represent the principal package types encountered in the sortation facility. Table 2 summarizes the dataset composition.

**Table 2:** Fine-tuning dataset composition by split and class.

Split	Images	Box	Envelope	Total annotations
Train	3,400	5,702	2,530	8,232
Validation	425	1,913	631	2,544
Test	425	2,368	565	2,933

Fine-tuning was initialized from COCO-pretrained RF-DETR Small weights and conducted on a commodity edge-class GPU. Training ran for a maximum of 30 epochs with early stopping enabled. The best checkpoint was selected at epoch 13 based on validation performance. A batch size of 2 was used with 8 gradient accumulation steps, yielding an effective batch size of 16. The learning rate was set to 1 × 10^−4^. Table 3 summarizes the fine-tuning configuration.

**Table 3:** Detector fine-tuning configuration.

Setting	Value
Base model	RF-DETR Small (COCO-pretrained)
Max epochs	30 (early stopping)
Selected checkpoint	epoch 13
Batch size	2
Gradient accumulation	8 steps (effective batch 16)
Learning rate	1 × 10^−4^
Inference precision	FP16
Inference input size	512 × 512

At inference time, the RF-DETR inference pipeline (Roboflow, 2025) internally resizes each input frame from its native 1,920 × 1,080 resolution to 512 × 512 pixels, performs detection at this reduced scale, and maps the resulting bounding box coordinates back to the original image dimensions. The confidence threshold is the only parameter whose value is task-specific: a stricter threshold $\tau_{\text{conf}}^{\text{dbl}}$ is applied to doubles detection to avoid false triggering of the two-line state machine, while throughput counting, reversal detection, and dwelling detection share a common, lower threshold τ_conf_. Both values were fixed during commissioning and held constant throughout the operational evaluation, as were all remaining parameters, which take a single value shared by all four monitoring tasks (Section 3.10). To reduce memory consumption and accelerate inference on the edge GPU, the model operates in half-precision (FP16) mode.

Following detection, two raw-detection bounding boxes $B_{d_{j}}^{t}$ and $B_{d_{k}}^{t}$ are compared through the Intersection over Union (IoU) and Intersection over Smaller area (IOS):


$$\begin{array}{l} \mathrm{IoU}\left(B_{d_{j}}^{t},B_{d_{k}}^{t}\right)=\frac{\left|B_{d_{j}}^{t}\cap B_{d_{k}}^{t}\right|}{\left|B_{d_{j}}^{t}\cup B_{d_{k}}^{t}\right|},\;\mathrm{IOS}\left(B_{d_{j}}^{t},B_{d_{k}}^{t}\right)=\frac{\left|B_{d_{j}}^{t}\cap B_{d_{k}}^{t}\right|}{\min\left(\left|\overset{t}{\underset{d_{j}}{B}}|,|B_{d_{k}}^{t}\right|\right)}. \end{array}$$ (7)

First, class-agnostic non-maximum suppression (NMS) eliminates redundant overlapping predictions when $\mathrm{IoU}\left(B_{d_{j}}^{t},B_{d_{k}}^{t}\right)\geq \tau_{\text{NMS}}$. Second, for the doubles detection task, a containment-based suppression step removes nested detections when $\mathrm{IOS}\left(B_{d_{j}}^{t},B_{d_{k}}^{t}\right)\geq \tau_{\text{IOS}}$. The additional suppression prevents spurious inner detections from triggering false doubles events when a single large package produces nested bounding boxes.

To restrict analysis to the relevant conveyor segment and discard detections from adjacent conveyors or background clutter, each induction point is associated with a predefined monitoring zone polygon Ƶ. Detections are retained only if a sufficient fraction of the bounding box area falls within this polygon. The zone filtering criterion is defined as follows.

** Definition 3.1 (Zone-Filtered Detection)**. *A detection* $d_{j}^{t}$ with bounding box $B_{d_{j}}^{t}$ *is retained if and only if*


$$\begin{array}{l} \frac{\left|B_{d_{j}}^{t}\cap Ƶ\right|}{\left|B_{d_{j}}^{t}\right|}\geq \tau_{\text{zone}}, \end{array}$$ (8)

*where* Ƶ *denotes the monitoring zone polygon, |·| denotes area, and τ_zone_ is the overlap threshold*.

### 3.4 Multi-object tracking

With detections established, the next step is to track each package across successive frames, building the trajectory histories that all downstream analysis modules consume. The system employs the ByteTrack algorithm (Zhang et al., 2022), implemented through the Supervision computer vision library (Roboflow, 2024). ByteTrack was selected for its computational efficiency and its effectiveness in scenarios characterized by linear object motion, the predominant motion pattern on conveyor systems. Unlike methods that discard low-confidence detections during association, ByteTrack incorporates all detection boxes into the matching process through its BYTE association strategy. This approach improves tracking continuity for packages that may temporarily receive lower confidence scores due to partial occlusion or motion blur.

The RTSP stream delivers frames at 30 FPS, but running inference on every frame would exceed the computational budget of the edge device, which concurrently executes all tracking and analysis functions. The end-to-end per-frame latency (detection, tracking, and all virtual sensor evaluations) is 67 ms, yielding a maximum sustainable rate of 15 FPS. To match this budget, the system applies frame decimation: every second frame is processed, yielding an effective rate of *f* = 15 FPS for throughput counting, doubles detection, and reversal detection. Dwelling detection does not use continuous tracking; once every 60 s it evaluates the detections already produced for the current processed frame, so it adds no inference load. All frames are buffered at the native rate for video recording purposes regardless of the decimation schedule.

When ByteTrack assigns a new track identity *i*, the system instantiates a trajectory record containing the first observed centroid ${\text{c}}_{i}^{0}$, a rolling buffer $H_{i}^{t}$ of the *N*_buf_ most recent centroid-timestamp pairs, and the corresponding timestamps. The centroid at each frame is computed as the geometric center of the tracked bounding box. Trajectory records that have not received an observation within a stale-track timeout *T*_stale_ are pruned to prevent unbounded memory growth and to avoid stale associations. These trajectory records provide the input to throughput counting, doubles detection, and reversal detection. Dwelling detection instead operates statelessly on the shared detections of the current processed frame at each scoring interval.

### 3.5 Throughput counting

Throughput counting is the first operational insight derived from trajectory data. It provides a continuous measure of the package flow rate at each induction point by detecting when tracked centroids cross a virtual trigger line. This trigger line is a horizontal line defined at a specific *y*-coordinate within the monitoring zone, oriented perpendicular to the direction of package flow. As a tracked package traverses the camera field of view, its centroid position is compared against the trigger line at each processed frame.

A package is counted when its centroid crosses the virtual trigger line for the first time. To prevent double counting from tracking noise or brief centroid oscillations near the line boundary, the system maintains a set $S_{\text{counted}}$ of track identities that have already been registered. Once a track ID is added to this set, subsequent crossings by the same track are ignored. This design ensures a strict one-to-one mapping between physical packages and counted events.

To produce temporally aggregated throughput metrics, the system computes deltas over a fixed aggregation interval *T*_agg_. At each interval, the current size of $S_{\text{counted}}$ is compared against the previous checkpoint value, and the difference is written to Amazon DynamoDB with a timestamp and device identifier. This delta-based approach provides a streaming throughput signal suitable for real-time dashboards and alerting without requiring post-hoc batch processing. Algorithm 2 formalizes the procedure.

Algorithm 2 Throughput counting via virtual trigger line. 
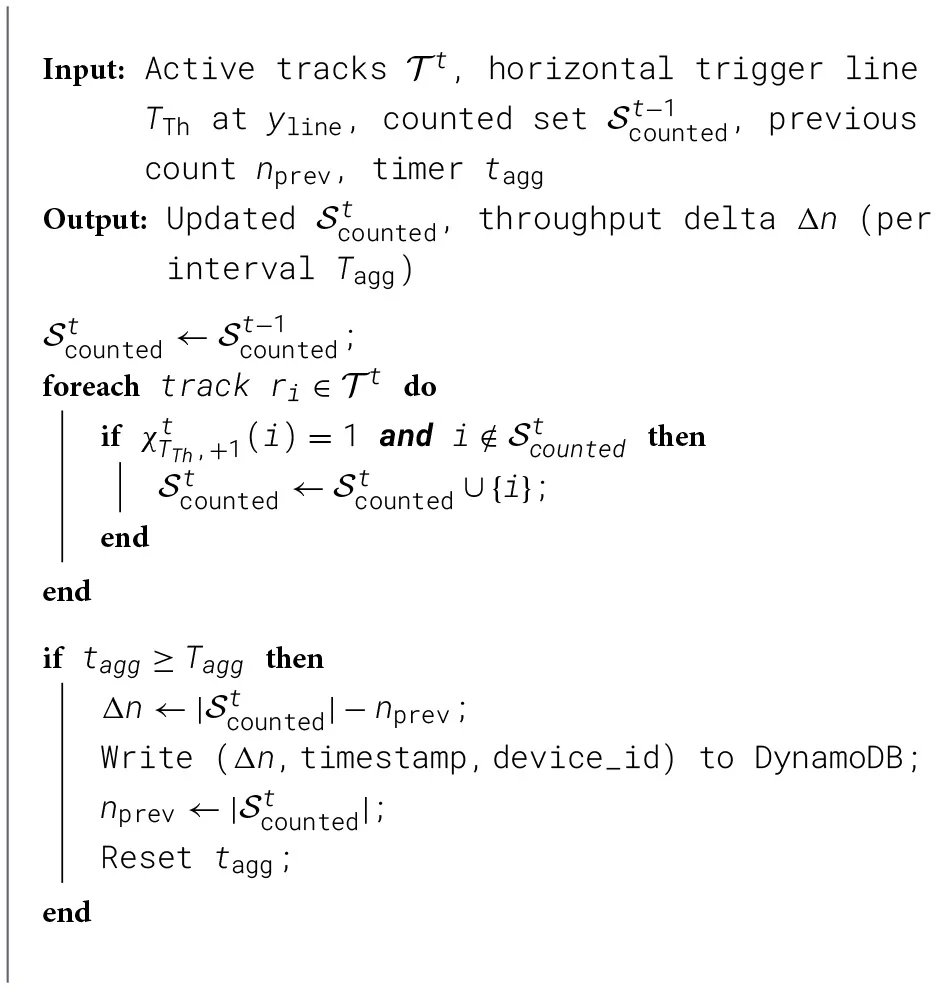

Using the directed line-crossing predicate in Equation 4, the counted-identity set and cumulative count after processed frame *t* are


$$\begin{array}{l} S_{\text{counted}}^{t}=S_{\text{counted}}^{t-1}\cup \left\{i\in T^{t}|\chi_{T_{\text{Th}},+1}^{t}\left(i\right)=1\right\},\\ \;C^{t}=|S_{\text{counted}}^{t}|. \end{array}$$ (9)

where $T^{t}$ is the active-track set. For the horizontal trigger line deployed in this study, Equation 4 reduces to the comparison of consecutive centroid *y*-coordinates used in Algorithm 2. For an aggregation interval [*t*_*k*−1_, *t*_*k*_], the reported throughput is


$$\begin{array}{l} \text{Δ}n_{k}=C^{t_{k}}-C^{t_{k-1}}. \end{array}$$ (10)

### 3.6 Doubles detection

Doubles detection represents the second trajectory-derived insight: identifying instances where two or more packages travel side by side and cross the induction point simultaneously. Such events are a primary cause of downstream jams and reversal events. When doubles are detected, the facility's quality control system reverses the belt direction to send the packages back into the reject bin. The doubles detection module employs a two-line crossing state machine that tracks each package through a sequence of spatial checkpoints to determine whether a detected double is subsequently reversed (sent into the reject bin) or escapes onto the sortation loop.

Two virtual trigger lines are defined within the monitoring zone: *L*_1_ positioned upstream and *L*_2_ positioned downstream toward the reject bin. To reduce false triggers caused by bounding box edges briefly intersecting *L*_1_, the bounding box is shrunk toward the center on all four sides before evaluating the crossing condition. A sliding window filter further requires that the crossing condition be satisfied in a majority of consecutive frames before a crossing event is confirmed. This temporal filtering eliminates transient detections caused by tracking jitter or momentary bounding box fluctuations.

Tracks that confirm *L*_1_ crossings within a temporal coincidence window form a doubles candidate. Each track in a confirmed doubles candidate enters a *monitoring* state in which the system awaits an *L*_2_ crossing. Two outcomes are possible. If the track crosses *L*_2_ within a timeout period *T*_doubles_, the event is classified as *reversed*, indicating that the doubled packages were detected by the facility's quality control mechanisms and sent back into the reject bin. If the timeout elapses without an *L*_2_ crossing, the event is classified as *escaped*, indicating that the doubled packages proceeded onto the sortation loop uncaught.

A spatial reassignment mechanism handles cases where a track is lost (for example, due to brief occlusion) and a new track appears in close proximity. If a new track ID is detected within a small spatial radius of the last known position of a monitored track, the monitoring state is transferred to the new track, preserving continuity of the doubles event. Specifically, ${\text{c}}_{j}^{\text{last}}$ denotes the most recent centroid stored for lost monitored track *j*. The spatial arrangement of *L*_1_ and *L*_2_ within the induction point is shown in Figure 1, and Algorithm 3 formalizes the procedure.

Algorithm 3 Doubles detection via two-line state machine. 
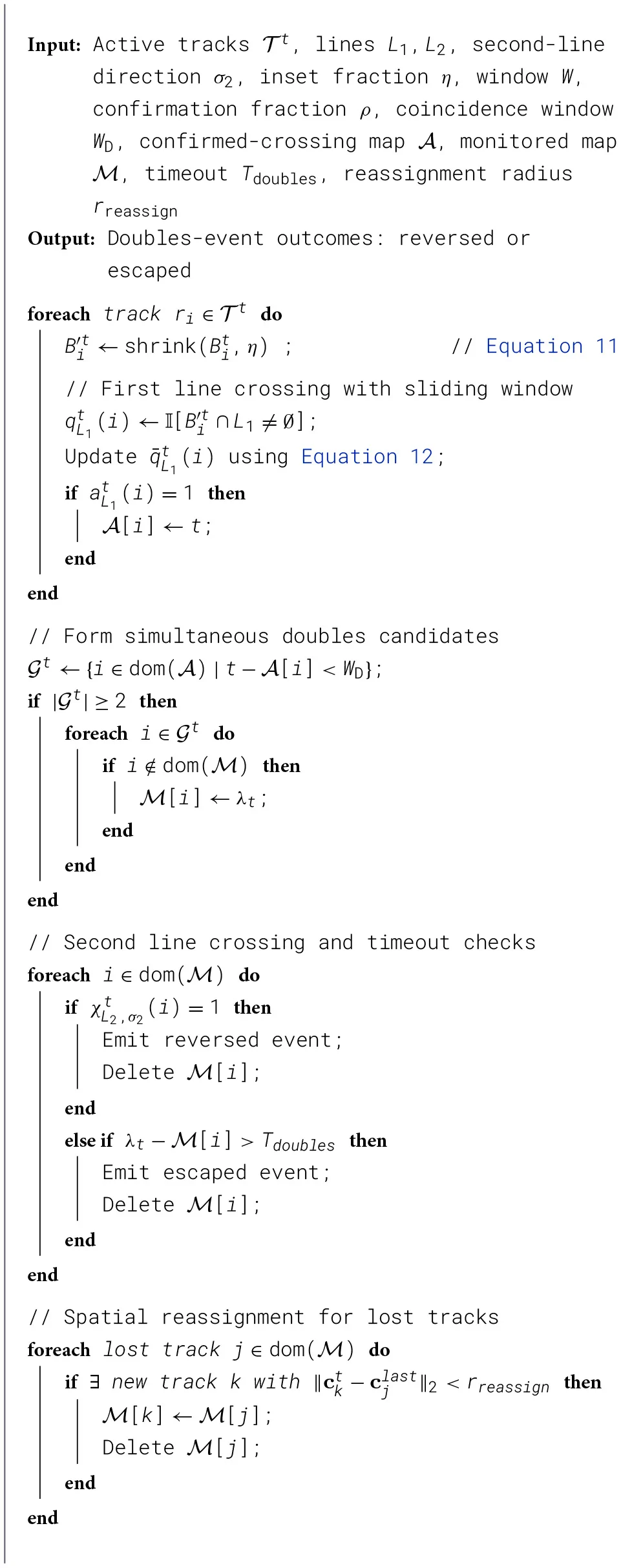

For a bounding box with width $w_{i}^{t}$ and height $h_{i}^{t}$, the inset box used for the first-line test is


$$\begin{array}{l} B_{i}^{\prime t}=\left(x_{i,\min}^{t}+\eta w_{i}^{t},y_{i,\min}^{t}+\eta h_{i}^{t},x_{i,\max}^{t}-\eta w_{i}^{t},y_{i,\max}^{t}-\eta h_{i}^{t}\right), \end{array}$$ (11)

where η is the inward inset fraction. Let $q_{L_{1}}^{t}\left(i\right)=1$ when $B_{i}^{\prime t}$ intersects *L*_1_ and $q_{L_{1}}^{t}\left(i\right)=0$ otherwise. The first-line crossing is confirmed when the proportion of positive observations in a window of *W* processed frames satisfies


$$\begin{array}{l} {\bar{q}}_{L_{1}}^{t}\left(i\right)=\frac{1}{W}\overset{t}{\underset{s=t-W+1}{\sum }}q_{L_{1}}^{s}\left(i\right),\;a_{L_{1}}^{t}\left(i\right)=\text{𝕀}\left[{\bar{q}}_{L_{1}}^{t}\left(i\right)\geq \rho \right], \end{array}$$ (12)

where ρ is the required confirmation fraction. Let $A^{t}$ map each confirmed *L*_1_ crossing to its confirmation time. The set of tracks confirmed within a coincidence window of *W*_D_ frames and the associated doubles predicate are


$$\begin{array}{l} G^{t}=\left\{i\in \mathrm{dom}\left(A^{t}\right)|t-A^{t}\left[i\right]<W_{\text{D}}\right\},\;D^{t}=\text{𝕀}\left[|G^{t}|\geq 2\right]. \end{array}$$ (13)

When *D*^*t*^ = 1, each track in $G^{t}$ enters the monitored map M with its entry timestamp λ_entry_(*i*). For a monitored track, the final outcome is


$$y_{i}=\{\begin{array}{ll} \text{reversed}, & \exists t^{\prime }:\chi_{L_{2},\sigma_{2}}^{t^{\prime }}(i)=1\;\wedge \;{\text{λ}}_{t^{\prime }}-{\text{λ}}_{\text{entry}}(i)\leq T_{\text{doubles}},\\ \text{escaped}, & {\text{λ}}_{t}-{\text{λ}}_{\text{entry}}(i)>T_{\text{doubles}}\text{ and no qualifying }L_{2}\text{ crossing occurs}. \end{array}$$ (14)

### 3.7 Reversal detection algorithm

The reversal detection algorithm is the core trajectory analysis component of the proposed system. It operates on per-package trajectory histories and combines displacement analysis with velocity-direction verification to determine whether a package has reversed its direction of travel on the conveyor. The fundamental insight is that a reversal manifests as a trajectory in which a package first travels a significant distance from its initial position, then begins moving back toward its point of origin with a velocity vector directed toward the start.

In practical terms, the algorithm checks whether a package moved far enough forward, stopped progressing, and is now actively traveling back toward where it started. The following notation formalizes these conditions for track *i*. Let ${\text{c}}_{i}^{0}\in {\mathbb{R}}^{2}$ denote its first observed centroid and ${\text{c}}_{i}^{t}\in {\mathbb{R}}^{2}$ its centroid at the current frame *t*. The Euclidean distance from the current position to the starting position is $d_{i}^{t}=||{\text{c}}_{i}^{t}-{\text{c}}_{i}^{0}{||}_{2}$. The maximum distance observed over the retained trajectory buffer is $d_{i,\max}^{t}=\underset{\left({\text{c}}_{i}^{s},{\text{λ}}_{s}\right)\in H_{i}^{t}}{\max}||{\text{c}}_{i}^{s}-{\text{c}}_{i}^{0}{||}_{2}$. The velocity vector is estimated over a lookback window of *k* frames and normalized by elapsed time:


$$\begin{array}{l} {\text{v}}_{i}^{t}=\frac{{\text{c}}_{i}^{t}-{\text{c}}_{i}^{t-k}}{{\text{λ}}_{t}-{\text{λ}}_{t-k}}. \end{array}$$ (15)

When $||{\text{v}}_{i}^{t}{||}_{2}>0$, the unit velocity vector is ${\hat{\text{v}}}_{i}^{t}={\text{v}}_{i}^{t}/||{\text{v}}_{i}^{t}{||}_{2}$, and the unit return vector (directed from the current position toward the start) is ${\hat{\text{r}}}_{i}^{t}=\left({\text{c}}_{i}^{0}-{\text{c}}_{i}^{t}\right)/||{\text{c}}_{i}^{0}-{\text{c}}_{i}^{t}{||}_{2}$.

All pixel-space thresholds in this work are hyperparameters tuned for a standardized camera installation (fixed mounting height, lens, and resolution); they are specified as multiples of the median observed package extent so that they transfer to other installations through a single measurable scale factor, as detailed in Section 3.10.

A package is classified as reversed when the following conditions are satisfied simultaneously. First, the package must have traveled at least a minimum displacement δ_min_ from its origin, ensuring that only meaningful displacements are considered. Second, the package must have returned at least δ_min_ from its farthest observed position, confirming that the package is no longer progressing along its original trajectory. Third, the velocity vector must be nonzero and aligned with the return direction, as measured by the cosine similarity ${\hat{\text{v}}}_{i}^{t}\cdot {\hat{\text{r}}}_{i}^{t}\geq \gamma$. This directional constraint prevents random positional jitter or brief pauses from being misinterpreted as reversals. Additionally, a minimum of *N*_min_ trajectory samples must have been accumulated before any reversal assessment is performed, providing sufficient observation history for reliable analysis. The complete procedure is formalized in Algorithm 4, and the geometric intuition underlying the vector analysis is illustrated in Figure 2.

Algorithm 4 Package reversal detection. 
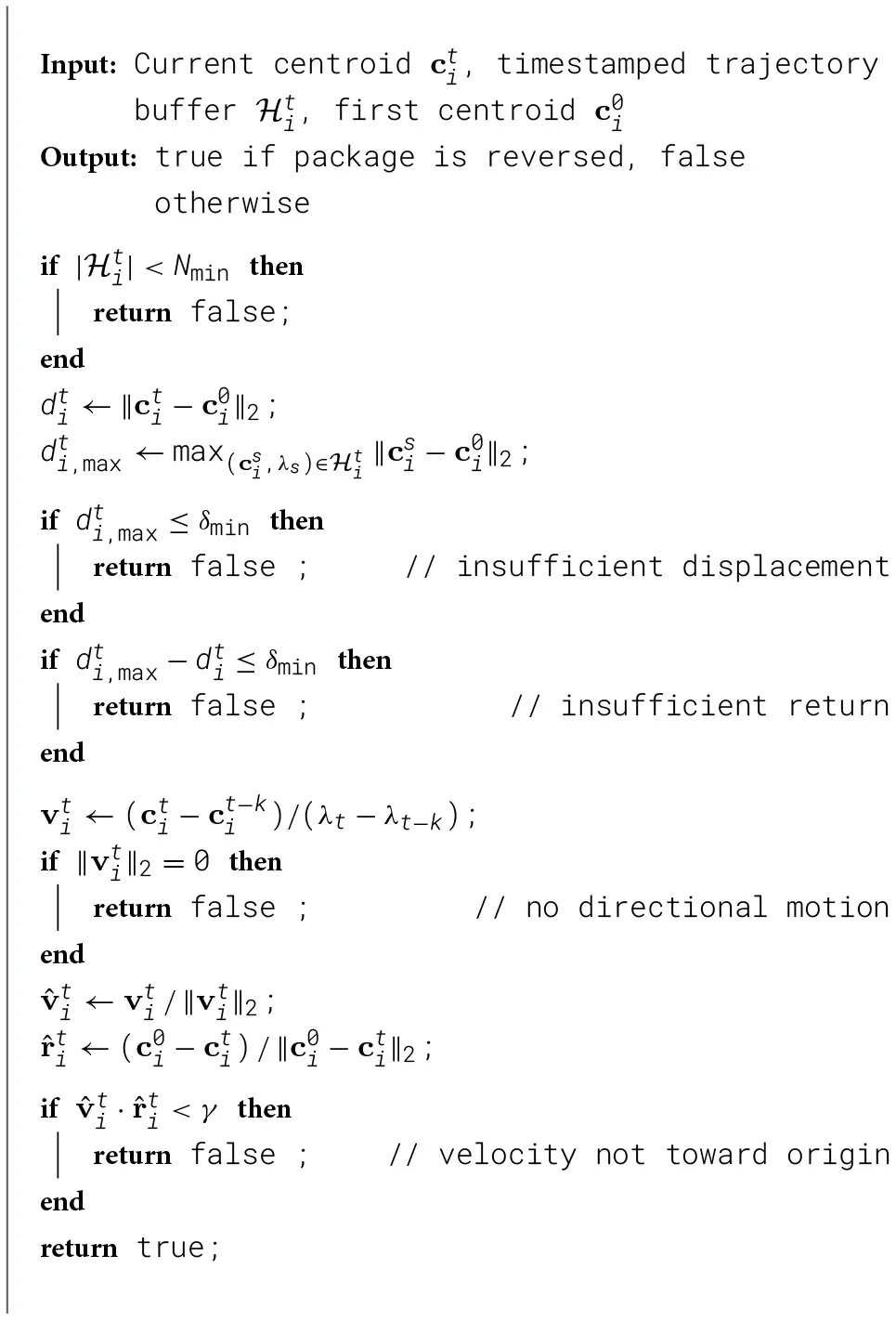

**Figure 2:**
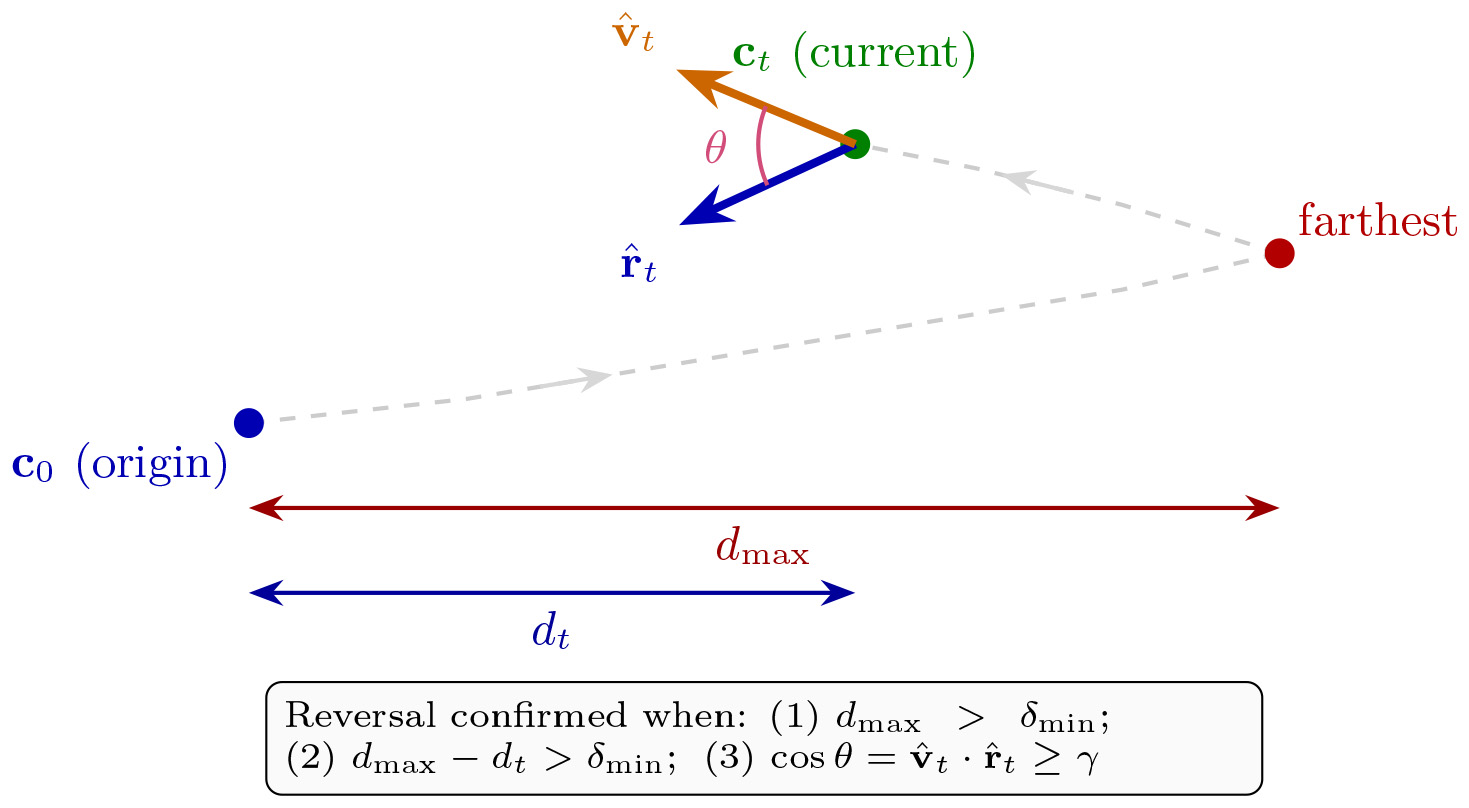
Geometric illustration of the reversal detection vector analysis. A package travels from its origin ${\text{c}}_{i}^{0}$ to a farthest point, then returns toward ${\text{c}}_{i}^{t}$. The velocity vector ${\hat{\text{v}}}_{i}^{t}$ and return vector ${\hat{\text{r}}}_{i}^{t}$ must be sufficiently aligned (cosine similarity ≥γ) for reversal confirmation.

** Definition 3.2 (Package Reversal)**. A tracked package *i* with $|H_{i}^{t}|\geq N_{\min}$ is classified as *reversed* at frame *t* if and only if:

1. $d_{i,\max}^{t}>\delta_{\min}$ (sufficient displacement),
2. $d_{i,\max}^{t}-d_{i}^{t}>\delta_{\min}$ (significant return),
3. $||{\text{v}}_{i}^{t}{||}_{2}>0$ and ${\hat{\text{v}}}_{i}^{t}\cdot {\hat{\text{r}}}_{i}^{t}\geq \gamma$ (velocity alignment).

The reversal detection logic is evaluated per-frame for every active trajectory. To govern the recording of video evidence, the system implements a three-state mechanism. Under normal operation (state N), the system continuously monitors all active trajectories while a rolling buffer retains the most recent frames at the native stream rate, enabling retrospective capture. When at least one reversed trajectory is detected, the system transitions to recording (state R): a pre-buffer of frames immediately preceding the reversal (*T*_pre_) is prepended to the recording, and all subsequent frames are appended in real time. When no active reversals remain, the system enters a post-recording phase (state P) and continues recording for an additional interval *T*_post_. If a reversal resumes during this interval, the system returns to state R; otherwise, the recording is finalized and enqueued for saving and upload, returning to state N. As a safety measure, any recording exceeding a maximum duration *T*_max_ is terminated to prevent excessive resource consumption.

### 3.8 Dwelling detection

Dwelling detection identifies packages that have accumulated in the reject bin. In principle, trajectory analysis can detect stationary packages by identifying centroids that remain fixed over time. However, because the reject bin is the designated destination for reversed packages, the problem reduces to detecting the *presence* of packages within the bin zone rather than analyzing their motion. The system therefore evaluates the zone state once per scoring interval *T*_dwell_ rather than requiring continuous tracking. This lightweight approach minimizes computational overhead while providing sufficient temporal resolution for detecting stalled packages, which by definition persist for extended durations.

At each scoring interval, the system takes the detections $D^{t}$ already produced by the shared RF-DETR pass for the current processed frame of the main loop (Algorithm 1) and applies zone filtering against the predefined dwelling zone polygon ${Ƶ}_{\text{dwell}}$. This polygon is defined independently of the general monitoring zone and delineates the specific spatial region where package stalling is of operational concern. For each retained detection, the system computes the bounding box centroid and evaluates whether it falls within ${Ƶ}_{\text{dwell}}$ using a point-in-polygon test. Algorithm 5 formalizes the procedure. Because dwelling detection reuses these shared detections, it does not perform an additional inference pass; its incremental cost is limited to one zone-overlap and point-in-polygon test per detection once every *T*_dwell_ seconds.

Algorithm 5 Dwelling detection via periodic zone scoring. 
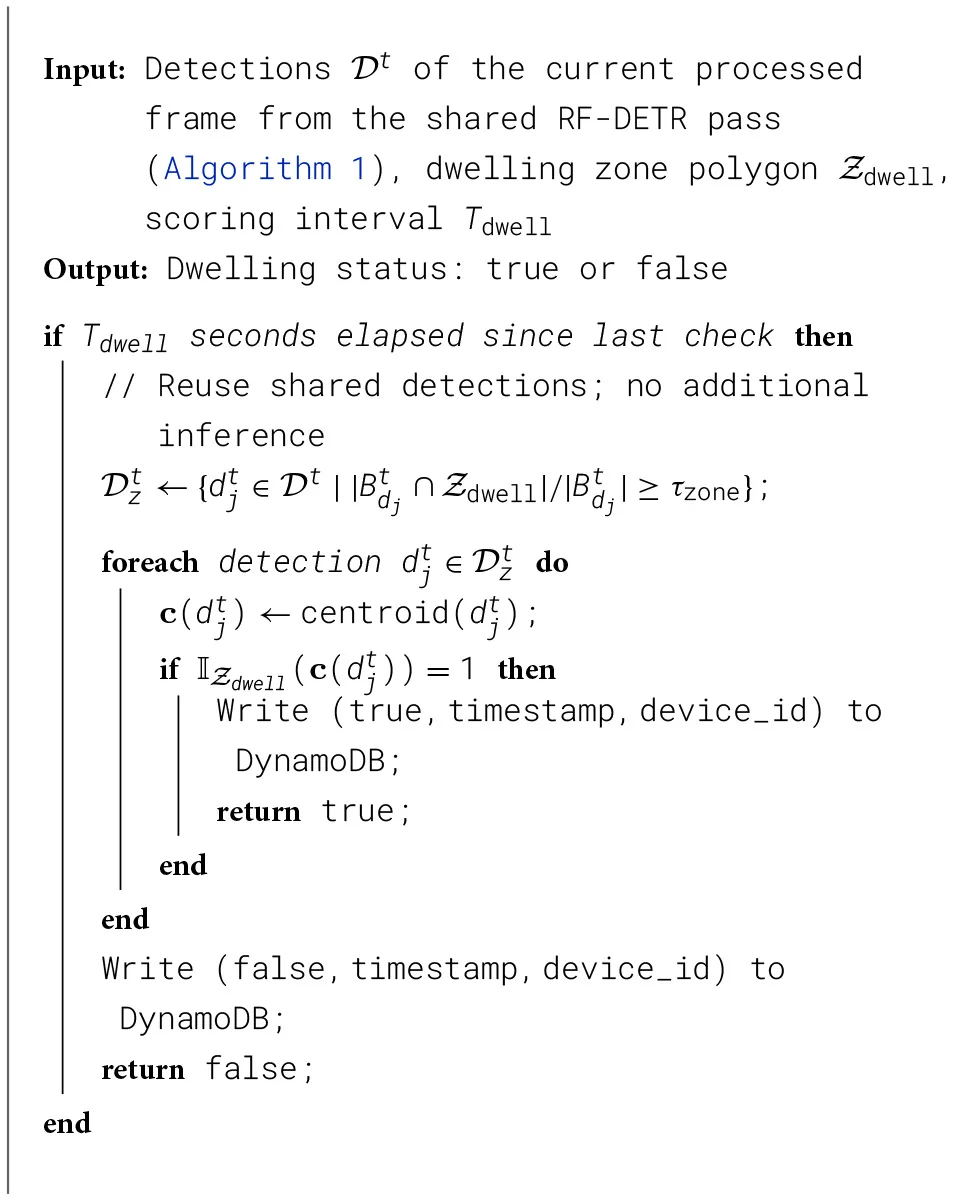

The output is binary and is formalized as


$$\begin{array}{l} d_{\text{dwell}}^{t}=\text{𝕀}\left[\exists d_{j}^{t}\in D_{z}^{t}\text{ such that }{\text{𝕀}}_{{Ƶ}_{\text{dwell}}}\left(\text{c}\left(d_{j}^{t}\right)\right)=1\right]. \end{array}$$ (16)

Thus, if any package centroid falls within the dwelling zone, the system reports dwelling = true; otherwise, it reports dwelling = false. The result, along with a timestamp and device identifier, is written to Amazon DynamoDB. Because this simple geometric test requires no tracking state, dwelling detection is fully stateless across scoring intervals and robust to track ID discontinuities.

### 3.9 Edge deployment architecture

The proposed system is deployed at induction points within a sortation facility. Each induction point is equipped with an overhead network camera that streams video at 1,920 × 1,080 resolution and 30 FPS over the Real-Time Streaming Protocol (RTSP). Each camera is positioned to provide a clear overhead view of the conveyor segment immediately upstream of the sorter interface.

Each edge device is equipped with an NVIDIA A1000 GPU operating within an 8 GiB VRAM budget. The use of FP16 inference is critical for fitting the RF-DETR Small model within this budget while maintaining real-time tracking and analysis throughput. The detection model and the privacy masking model (described below) share the GPU and together consume under 1 GB of GPU memory in operation. Explicit memory management through periodic cache clearing prevents memory fragmentation during continuous operation.

AWS IoT Greengrass V2 (Amazon Web Services, 2023) orchestrates software deployment and lifecycle management on the edge device. Greengrass provides over-the-air deployment of application updates and configuration changes. Each device reads a device identifier from the local Greengrass configuration file at startup. Recorded event video clips are uploaded to Amazon S3 for persistent storage and subsequent review, while structured event data (including throughput counts, doubles detection outcomes, reversal event metadata, and dwelling status) is written to Amazon DynamoDB for querying and dashboard integration.

To comply with workplace privacy policies, the system implements a privacy-preserving pipeline that ensures no personally identifiable information is stored on the edge device or transmitted to cloud storage. A separate RF-DETR Medium model (Robinson et al., 2025), trained on a general-purpose person detection dataset, runs on each recorded event to detect persons in the camera field of view. If any person is detected within the video zone polygon with sufficient bounding box overlap, the entire frame receives Gaussian blur via the OpenCV library (OpenCV Team, 2025) before being written to disk or uploaded. When no persons are detected, regions outside the predefined monitoring zone polygon are blurred while the conveyor area is preserved at full resolution. Under no circumstances is unfiltered video stored or transmitted. The privacy and cloud components of the edge deployment architecture are shown in Figure 3.

**Figure 3:**
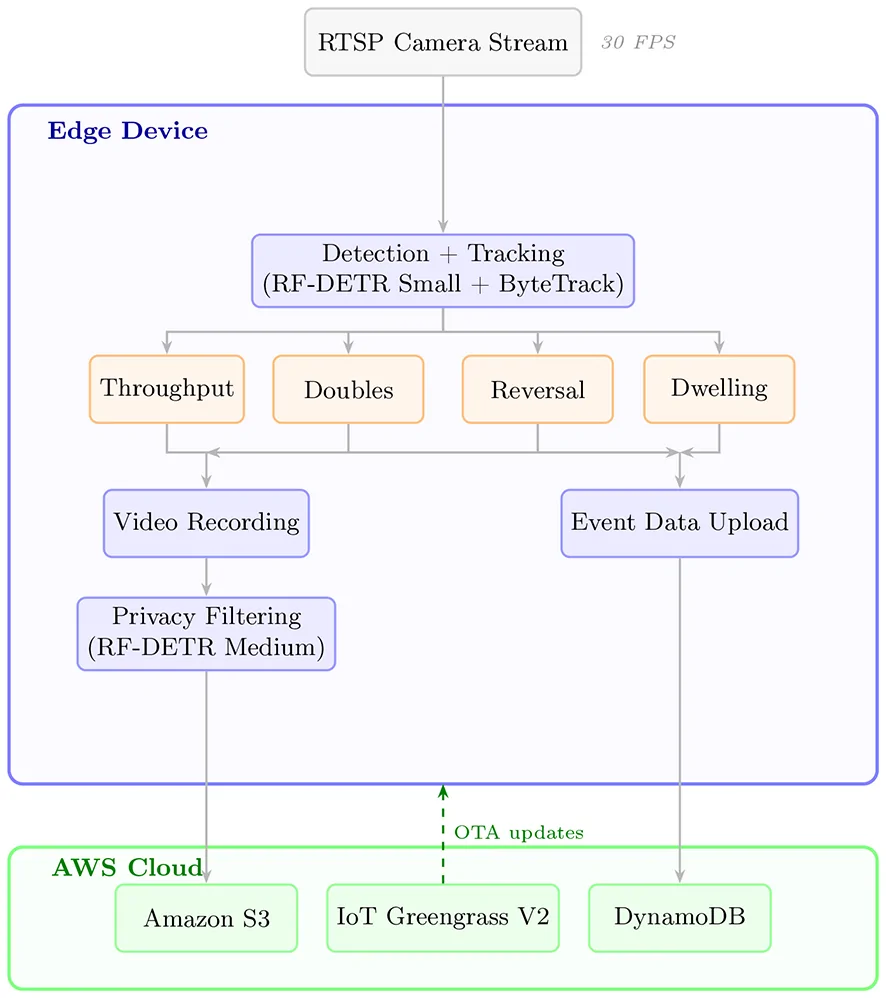
General architecture and edge deployment of the proposed framework. Each edge device performs detection, tracking, trajectory analysis, and privacy filtering independently. Video evidence is uploaded to Amazon S3 after privacy filtering, and structured event data is written to Amazon DynamoDB. AWS IoT Greengrass V2 manages over-the-air updates to the device.

### 3.10 Parameter selection and reproducibility

The behavior of the monitoring algorithms is governed by a set of detection, geometric, temporal, and kinematic parameters introduced symbolically in Sections 3.3–3.8: the detection-confidence thresholds, the NMS and containment-suppression thresholds, the zone-overlap threshold, the bounding-box inset fraction, the sliding-window length and confirmation ratio, the coincidence window, the doubles timeout, the spatial reassignment radius, the minimum trajectory-sample count, the minimum displacement, the velocity lookback, the direction-alignment threshold, the trajectory buffer length, the stale-track timeout, and the aggregation, scoring, and video-buffer intervals. The numerical settings used in the deployment are specific and proprietary to the operating environment and cannot be released. To support independent reproduction, we instead specify the quantity that each parameter is defined relative to, the ordering constraints that any valid configuration must satisfy, and the procedure by which the deployed values were obtained.

#### 3.10.1 Dimensional basis of each parameter group

The parameters fall into four groups with different transfer properties across installations. (i) The confidence, NMS, containment, zone-overlap, confirmation-ratio, bounding-box inset, and direction-alignment parameters are dimensionless quantities on [0, 1]. They are independent of image resolution, camera geometry, and conveyor speed, and therefore transfer directly to another installation without rescaling. (ii) The displacement and reassignment parameters are the only pixel-space quantities. Rather than absolute pixel counts, each is specified as a multiple of the median package bounding-box extent observed at the installed camera geometry, which couples them to resolution, mounting height, and lens field of view through a single measurable scale factor. On a new installation, both are recovered by measuring this median extent and applying the same multiple. Equivalently, they may be expressed as a fraction of the image width, in which case they rescale linearly with the capture resolution. (iii) The frame-count parameters (sliding window, coincidence window, velocity lookback, minimum sample count, trajectory buffer length) are specified in processed frames and are therefore tied to the processing rate *f*; they are converted to and from physical durations by dividing by *f*, so that a deployment running at a different rate preserves the intended temporal extent. (iv) The remaining timing parameters are specified in seconds and are independent of both resolution and processing rate.

#### 3.10.2 Ordering constraints

The following relations constrain the parameters independently of their numerical values, and are the relations that a reproduction should preserve. The doubles confidence threshold is strictly greater than the threshold shared by the other tasks, because a false detection there propagates into the two-line state machine. The containment threshold is strictly greater than the NMS threshold, since containment suppression targets fully nested boxes rather than partially overlapping ones. The confirmation ratio requires a strict majority of the sliding window. The sliding-window length is set so that the window spans at least the time a package traveling at nominal belt speed needs to traverse its own inset bounding box across the trigger line, which is the shortest interval over which a genuine crossing remains observable. The doubles timeout exceeds the nominal transit time from *L*_1_ to *L*_2_ at nominal belt speed, so that a package that does reverse is not timed out before reaching *L*_2_. The minimum displacement exceeds the centroid jitter scale of the detector, measured as the positional spread of a stationary package across consecutive frames, while remaining small relative to the length of the conveyor segment in view, so that reversals are detected before the package leaves the field of view. The minimum trajectory-sample count exceeds the velocity lookback, so that a velocity estimate is always available when a reversal assessment is made. The spatial reassignment radius is of the order of a single package extent, so that monitoring state transfers only between observations that cannot correspond to two distinct packages. The stale-track timeout exceeds the longest expected residence time of a package within the field of view. The trajectory buffer is long enough to span the reversal assessment horizon at the processing rate. The maximum recording duration exceeds the sum of the pre- and post-event buffers. The aggregation and dwelling-scoring intervals are determined by the reporting cadence required by facility operations rather than by any perception consideration, and may be set freely without affecting event-detection behavior.

#### 3.10.3 Selection procedure

All parameters were selected once, during commissioning and prior to the operational evaluation, by sweeping candidate values over a library of recorded video clips captured from the deployed camera installation. The clips were drawn from multiple induction points and multiple shifts, and were chosen to include both nominal single-package flow and the anomaly conditions of interest. Each candidate configuration was replayed offline through the same pipeline used in production, and the events it produced were compared against manual review of the same clips. Candidates were ranked by the number of false alerts they generated, subject to not introducing missed events relative to the manual review, and the dimensionless thresholds were swept over their full admissible interval while the pixel-space and temporal parameters were swept around the values implied by the ordering constraints above. The selected configuration was then frozen and used unchanged throughout the evaluation reported in Section 4; no retuning was performed between commissioning and evaluation, and no per-camera or per-induction-point adjustment was applied.

#### 3.10.4 Task-specific versus shared parameters

The detection confidence threshold is the only parameter whose value differs between monitoring tasks: a stricter threshold is applied to doubles detection, while throughput counting, reversal detection, and dwelling detection share a common, lower threshold. All remaining parameters take a single value shared by all four monitoring functions. Both confidence values, and every shared parameter, were fixed by the commissioning procedure above and held constant across all four tasks for the entire evaluation.

## 4 Results

This section evaluates the tracking and trajectory analysis system across its full operational scope. Because all monitoring capabilities rely on a shared detection and tracking backbone, the evaluation begins with the detection model (Section 4.1) and then assesses each downstream output in turn: the trajectory-derived outputs of throughput counting (Section 4.2), doubles detection (Section 4.3), and reversal detection (Section 4.4), followed by the detection-derived dwelling output (Section 4.5).

Each subsection describes the evaluation methodology and presents quantitative results. The reported metrics vary by task based on ground-truth availability: throughput counting reports accuracy against manual counts; doubles and reversal detection report precision on a large sample of system-generated alerts, complemented by a recall estimate on a smaller interval of continuously annotated video, because recall requires exhaustive manual review of continuous footage; dwelling detection reports precision, recall, F1, and accuracy since ground truth can be established from static image snapshots.

### 4.1 Detection model performance

Table 4 presents the per-class detection performance on the validation and test splits. On the validation set, the model achieved an overall mAP@50 of 96.1% with a precision of 98.1% and a recall of 90.3%, yielding an F1 score of 94.0%. On the held-out test set, the model attained an overall mAP@50 of 96.5% with a precision of 98.3% and a recall of 94.7%, yielding an F1 score of 96.5%. As noted in Section 3.3, the test images share cameras and sites with the training images, so these figures characterize performance under related acquisition conditions and do not independently establish cross-camera or cross-site generalization.

**Table 4:** RF-DETR small detection performance after fine-tuning.

Split	Class	mAP@50:95	mAP@50	Precision	Recall	F1
Validation	Box	0.627	0.952	0.977	0.898	0.936
	Envelope	0.755	0.970	0.983	0.911	0.946
	All	0.691	0.961	0.981	0.903	0.940
Test	Box	0.673	0.982	0.993	0.945	0.968
	Envelope	0.697	0.948	0.923	0.936	0.930
	All	0.685	0.965	0.983	0.947	0.965

Figure 4 compares the F1 score of our fine-tuned RF-DETR Small against several real-time detection architectures benchmarked by Halawa et al. (2025) on a similar conveyor package detection task and dataset. Although dataset-specific differences in annotation conventions and package distributions preclude a strict controlled comparison, the comparison is presented as contextual reference rather than as a performance ranking. Our model achieves an F1 of 0.965 on our test set, while Halawa et al. report F1 values of 0.86–0.87 for RTMDet variants, 0.77–0.83 for YOLO10/12 variants, and 0.82 for RT-DETR on their dataset. Because the datasets, annotation conventions, training protocols, and evaluation splits differ, and because our own test set shares acquisition conditions with the training data (Section 3.3), these figures place our result in the context of reported performance on related conveyor workloads but do not establish the architectural superiority of RF-DETR Small over the compared detectors.

**Figure 4:**
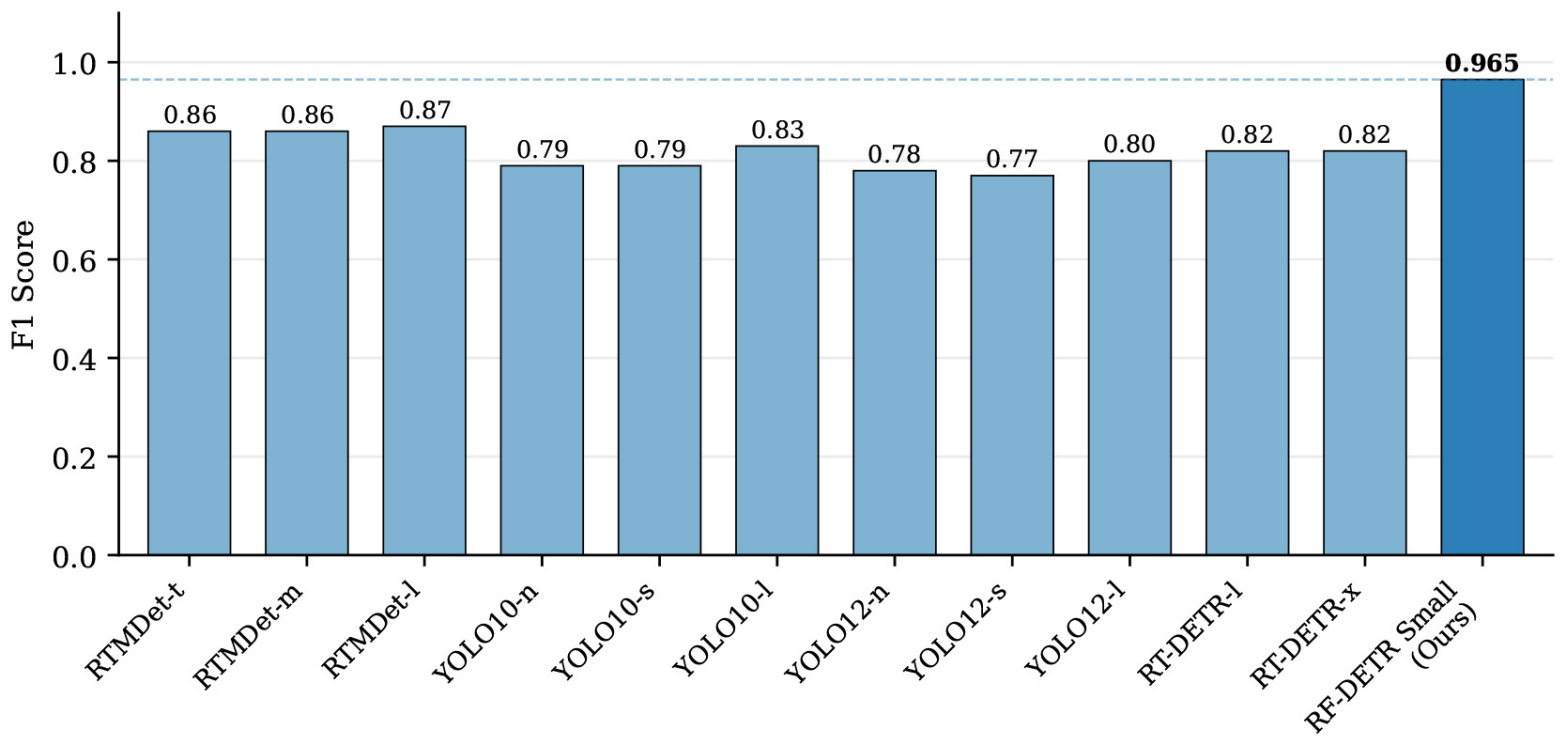
F1 score comparison of real-time object detection models on conveyor package detection. Baseline results from Halawa et al. (2025); our fine-tuned RF-DETR Small (rightmost, dark) is evaluated on a different dataset. The comparison is contextual and does not constitute a controlled common-dataset benchmark.

### 4.2 Throughput counting evaluation

Throughput counting validates the most fundamental capability of the tracking pipeline: accurately registering each package that traverses the induction point. The module was evaluated by comparing automated counts against manual ground truth on five production video sequences, each approximately five minutes in duration and captured from active induction points during standard operational hours. A human annotator independently counted all packages passing through the monitoring zone to establish ground truth. The system count increments when a tracked package trajectory crosses the virtual trigger line.

Table 5 presents the per-video results. The system achieved 98.05% counting accuracy across 513 ground truth packages, with two videos attaining perfect 100% accuracy. All counting errors were undercounts; no overcounting occurred in any video.

**Table 5:** Throughput counting accuracy across five production videos.

Video	Duration	Ground truth	System count	Accuracy (%)
1	~5 min	98	96	97.96
2	~5 min	112	112	100.00
3	~5 min	87	87	100.00
4	~5 min	105	102	97.14
5	~5 min	111	106	95.50
Total	~25 min	513	503	98.05

### 4.3 Doubles detection evaluation

Doubles detection builds on the same tracked trajectories to determine whether two or more packages were inducted simultaneously. The two-line state machine (Section 3.6) classifies each detected doubles event into one of two outcomes: the packages are reversed by the conveyor control system, or they escape past the reversal mechanism and continue downstream. The evaluation assessed whether the system correctly identified genuine doubles events for each outcome category.

A total of 500 production events were reviewed: 250 with a reversed outcome and 250 with an escaped outcome. Each event was manually verified against recorded video evidence. A false positive is defined as an event flagged as a doubles occurrence when only a single package was present.

Table 6 summarizes the results. The reversed category achieved 99.6% precision, with only 1 false positive out of 250 reviewed events. The escaped category achieved 92.4% precision, with 19 false positives. Overall, the two-line state machine achieved 96.0% precision across both outcome categories (*n* = 500).

**Table 6:** Doubles detection precision by event outcome.

Outcome	Reviewed	Correct	FP	Precision (%)
Reversed	250	249	1	99.6
Escaped	250	231	19	92.4
Overall	500	480	20	96.0

Because precision characterizes only the correctness of issued alerts, event recall was estimated separately. Approximately 6 h of continuous video from multiple induction points were manually reviewed, and every genuine doubles induction in this footage was annotated independently of the system output. The annotated events were then matched against the alerts produced by the deployed system over the same interval. Of the 30 annotated doubles events, 20 were detected, giving a recall of 66.7% (95% Wilson confidence interval 48.8%–80.8%). All 10 missed events were perception-level failures rather than failures of the two-line state machine: they involved two visually similar envelopes, or an envelope and a box, stacked or overlapping on top of each other so closely that the detector produced a single bounding box for both packages. In these configurations no second detection is available to the downstream logic, so the miss cannot be recovered by trajectory analysis alone.

This trade-off between precision and recall reflects a deliberate design choice. A doubles alert can trigger a belt reversal and operator intervention, so false alarms carry a direct operational cost; the module is therefore configured for high precision through the stricter doubles confidence threshold and the containment-based suppression of nested boxes (Section 3.3), which also suppresses some genuine but heavily overlapping second packages. The reported recall should accordingly be interpreted as a lower bound on what the trajectory logic can achieve given perfect detection, and it identifies the separation of stacked, visually similar packages as the principal open problem for vision-based doubles detection.

### 4.4 Reversal detection evaluation

Reversal detection applies trajectory displacement and velocity analysis (Algorithm 4) to identify packages that change direction on the conveyor. To evaluate precision, a sample of 500 reversal events was manually reviewed against recorded video evidence post deployment. Each event was assessed to determine whether the flagged trajectory displacement corresponded to an actual physical direction change on the conveyor.

Table 7 presents the precision results. Of the 500 reviewed events, 483 were confirmed true positives and 17 were classified as false positives, yielding an operational precision of 96.6%. Of these 17, 16 were operator-induced reversals (a worker manually pulling a package backward), which the algorithm correctly detected as direction changes but which fall outside the intended equipment-malfunction alerting scope. The raw algorithmic precision is therefore 99.8% (499/500).

**Table 7:** Reversal detection precision based on manual review of 500 events.

Reviewed	TP	FP	Precision (%)
500	483	17	96.6

Reversal recall was estimated on the same approximately six hours of continuously annotated video used for doubles detection, in which every genuine package reversal was annotated independently of the system output. Of the 27 annotated reversals, 24 were detected, giving a recall of 88.9% (95% Wilson confidence interval 71.9%–96.1%). Unlike the doubles misses, the three missed reversals were not caused by detection failures: the packages were detected and tracked, but the trajectory did not satisfy the reversal conditions of Algorithm 4 at the configured thresholds. These cases therefore represent genuine misses of the reversal logic and indicate that the displacement and direction-alignment criteria leave room for improvement.

### 4.5 Dwelling detection evaluation

Dwelling detection applies periodic zone scoring to identify packages that have accumulated in the reject bin. Because the system evaluates zone presence on the detections of a single processed frame per scoring interval (Section 3.8), the evaluation validates per-frame presence detection rather than temporal persistence. A total of 500 snapshot images were captured and manually labeled as either “dwelling” (package present in zone) or “clear” (zone empty). Of these, 182 were actual dwelling events and 318 were clear.

Table 8 summarizes the performance metrics. The perfect precision (100%) means that every dwelling alert issued by the system corresponded to a genuine package presence, imposing zero false alarm burden on operators. The recall of 96.15% reflects 7 missed dwelling events out of 182.

**Table 8:** Dwelling detection performance metrics (*n* = 500).

Metric	Value
Precision	100.00%
Recall	96.15%
F1 score	98.04%
Accuracy	98.60%

## 5 Discussion

### 5.1 Computational efficiency of single-pass tracking

The shared detection-and-tracking backbone yields substantial computational savings compared to deploying independent pipelines for each monitoring task. With a measured per-frame latency of 67 ms (Section 3.4), the single inference pass consumes the majority of the computational budget; the four downstream modules collectively add negligible overhead because they perform only lightweight geometric operations (trigger-line intersections, state-machine transitions, and vector dot products on the already-computed trajectories, and point-in-polygon tests on the already-computed detections for dwelling detection). Running independent detection-plus-tracking stacks for each task would multiply the GPU memory and inference compute required, scaling poorly on resource-constrained edge hardware. By contrast, prior logistics monitoring systems (Halawa et al., 2025; El-sayed et al., 2022) deploy separate detection pipelines per task or rely on task-specific physical sensors, incurring proportional scaling costs.

At the fixed input resolution and 15 FPS processing schedule used in this deployment, the RF-DETR Small inference workload is expected to remain largely independent of the number of packages present in a frame because each selected frame is processed by a single, fixed-cost detector pass. The multi-threaded design further isolates GPU inference from frame capture, video encoding, cloud upload, and logging. For deployments with higher operational demand, additional RAM can support larger frame, event-video, and work queues, while additional CPU cores can increase the available capacity for these concurrent non-inference stages. The lightweight trajectory and virtual-sensor computations operate on the detections already produced by this shared pass and do not require additional model inference. Although the present deployment sustained 15 FPS under normal facility traffic, peak-density stress testing remains future work; at higher densities, the primary concern is expected to be visual occlusion and track continuity rather than detector throughput.

These efficiency properties are confirmed in production. Measured over an approximately 19-h window comprising 59,791 telemetry samples, each device sustains the target processing rate of 15 FPS while consuming a median resident memory of 2.0 GB (95th percentile 2.1 GB) against a 6 GiB container allocation, under 1 GB of GPU memory, and approximately 1.2 CPU cores. Processed throughput stayed at or above 14.5 FPS in 99% of samples, and no event clips were dropped during the measurement window, indicating a negligible frame-drop rate under normal facility load. This low and uniform footprint leaves substantial headroom on the edge hardware, allowing a single device to serve several induction points rather than one.

The shared backbone also simplifies maintenance. A single model update simultaneously improves all four tasks without requiring task-specific model management, and configuration changes propagate through a unified parameter file deployed via AWS IoT Greengrass V2. This operational simplicity is a practical prerequisite for scaling to additional monitoring points.

### 5.2 Virtual sensors vs. physical sensors

The system instantiates what we term *virtual sensors*: software-defined trigger lines and detection zones that can serve as an alternative or complement to dedicated physical sensor hardware for selected monitoring functions. Compared to RFID-based scanning approaches (Ning et al., 2022) and conventional photoeye arrays used in sortation facilities, this paradigm offers several structural advantages.

First, virtual sensors substantially reduce signal jittering. Physical photoeyes generate binary beam-break events. When a package oscillates near the beam boundary, a single physical event can produce multiple triggers in rapid succession. The CV system tracks unique package identities through persistent track IDs, inherently deduplicating events that photoeyes cannot distinguish. Analogous digital noise (centroid oscillations near trigger lines) is addressed through sliding-window filtering and minimum-sample thresholds.

Second, virtual sensors produce richer metadata than binary signals. Each detection event is associated with bounding-box geometry, trajectory history, timestamps, and video evidence. This information enables downstream analytics (such as trend analysis and correlation with upstream process variables) that are unattainable from sensor logs alone.

Third, virtual sensors are reconfigurable without hardware changes. Adjusting the position of a trigger line or the geometry of a detection zone requires only a coordinate update in the configuration file, deployable over the air within minutes. Adding a new monitoring task to an existing camera requires no physical installation, only the definition of new post-processing logic operating on the same shared detections.

Fourth, a single camera serves multiple purposes simultaneously. The same video feed and the same detection outputs drive throughput counting, doubles detection, and reversal detection through the shared trajectories, and dwelling detection directly through the shared detections. This consolidation can reduce the number of dedicated photoeye arrays required for the monitoring functions considered here and, with it, per-station instrumentation.

These advantages are structural and follow from the design of the framework; they have not been established through a direct comparison with physical sensors. The present study does not measure physical sensors side by side with the vision system in terms of accuracy, latency, reliability, cost, or failure behavior, and cameras introduce failure modes of their own, such as occlusion, lighting changes, lens contamination, and detector errors, that differ from those of photoeyes. The framework is therefore best regarded as a potential alternative or complement to physical sensors for selected non-safety-critical monitoring functions. Safety-critical interlocks and control functions that currently rely on physical sensors are outside the scope of this work, and a controlled side-by-side comparison is an important direction for future research.

### 5.3 Edge deployment lessons

Continuous operation at induction points has yielded several practical insights. The multi-threaded architecture proved essential for maintaining real-time throughput. By decoupling frame capture, GPU inference, video encoding, cloud upload, and log management into independent threads, the system ensures that no single I/O bottleneck disrupts inference. Without this separation, network latency during S3 uploads would introduce inference delays, and disk I/O during video encoding would cause frame drops.

The privacy-first design, which applies person detection and Gaussian blur on-device before any data leaves the edge, was a non-negotiable requirement for production deployment. It did not introduce measurable latency overhead given the existing GPU headroom. Over-the-air updates via AWS IoT Greengrass V2 enabled rapid iteration, with algorithm improvements and threshold adjustments deployed within hours of validation and without requiring physical access to the device.

The sustained operation of edge devices processing video continuously at 15 FPS confirms that the edge hardware and software stack are robust under continuous industrial workloads.

### 5.4 Transferability

The operational evaluation was conducted at a single facility, on a single conveyor type, with a standardized camera installation and empirically tuned thresholds. Performance under different camera geometries, conveyor configurations, package distributions, and operating conditions has therefore not yet been demonstrated. Within this limit, the framework is modular and potentially transferable because it is built on two domain-agnostic capabilities: object detection and multi-object tracking. In principle, any environment where packages (or other objects) move along a predictable path under a fixed camera could use the same pipeline. Curved conveyors, merge points, and split junctions are expected to require redefinition of the virtual sensor geometries (trigger-line angles, zone polygons) rather than architectural changes, although the monitoring logic itself has not been validated in these configurations. Similarly, different package distributions (e.g., predominantly envelopes, irregular parcels, or polybags) primarily affect the detection model, which can be retrained on site-specific data without modifying the downstream trajectory analysis logic; whether the downstream thresholds then transfer without recalibration remains to be tested.

The virtual sensor abstraction further supports transferability: adding a new monitoring task (e.g., orientation classification or label-side verification) requires only defining a new geometric or kinematic condition over the existing tracked trajectories, with no additional hardware or inference passes. The primary constraint on transferability is the manual tuning of pixel-space thresholds, which assumes a standardized camera installation. Facilities with heterogeneous camera mounting heights or lens configurations would require per-station calibration or an automated threshold adaptation mechanism.

### 5.5 Limitations

Several limitations merit acknowledgment. First, the results are obtained from a single conveyor type, and empirical validation across diverse facility types, conveyor topologies, and operational conditions has not yet been conducted; the shared detection backbone is also a single point on which all four tasks depend. Relatedly, the detection dataset was split randomly at the image level, so the same cameras and sites, including the evaluation facility, are represented in both the training and test sets. The reported detection metrics and the operational results therefore reflect in-distribution acquisition conditions; cross-camera and cross-site generalization would require a grouped evaluation in which entire cameras or sites are held out, which is left to future work. Second, the system relies on empirically tuned thresholds that assume a standardized camera installation and may require recalibration under different camera geometries or package distributions; no long-term study of performance degradation due to model drift has yet been performed. Third, recall for doubles and reversal detection is estimated on approximately six hours of continuously annotated video comprising 30 doubles and 27 reversal events. These sample sizes are small relative to the 500-event precision evaluations, and the resulting confidence intervals are correspondingly wide; a larger continuous annotation campaign is needed to estimate false-negative rates more precisely. The doubles recall of 66.7% is limited mainly by stacked or overlapping packages that the detector resolves as a single object, and the three missed reversals indicate that the reversal criteria can be improved. Accordingly, the doubles module should currently be regarded as a high-precision alerting function rather than an exhaustive detector of all doubles inductions. Fourth, the tracking stage is not evaluated independently: identity switches, track fragmentation, and trajectory continuity were not measured against annotated ground-truth tracks. Because throughput counting, doubles detection, and reversal detection all consume ByteTrack trajectories, tracking errors can propagate to each of these outputs, for example as a double count after an identity switch, a lost doubles monitoring state, or a truncated trajectory that prevents a reversal from being recognized. The task-level accuracy, precision, and recall figures capture the end-to-end effect of such errors on the monitored events but do not isolate their frequency. A dedicated tracking evaluation using standard multi-object tracking metrics on annotated conveyor sequences is left to future work.

Detection errors can propagate through the shared pipeline because tracking and virtual-sensor evaluation depend on the detector outputs. A missed detection can create a gap in a trajectory or prevent a line-crossing event from being observed; a false detection can initiate a spurious track; and bounding-box localization error can shift the centroid used by trigger lines, zones, and velocity calculations. The held-out detection results establish strong aggregate performance, but they do not isolate the effect of these error modes on each downstream monitoring task. ByteTrack association, per-track counted-ID sets, temporal confirmation, and spatial reassignment reduce the impact of some transient errors, but they do not correct persistent detector failures. A controlled sensitivity analysis that relates detection errors to throughput, doubles, reversal, and dwelling outputs is therefore an important direction for future work.

The current pipeline incorporates limited safeguards against short-term visual disruption. ByteTrack retains low-confidence detections during association to improve continuity through partial occlusion and motion blur, while the doubles module applies temporal confirmation and spatial reassignment to reduce the effect of transient track loss. These mechanisms do not constitute a controlled robustness evaluation. The data were collected under a standardized camera installation, and performance under substantial illumination changes, prolonged or dense occlusions, and packages with atypical shapes or deformation has not been independently quantified. Such conditions may reduce detector confidence, interrupt track identity, or alter bounding-box geometry used by the virtual sensors. Future validation should therefore include condition-stratified test data and, where needed, targeted data augmentation or site-specific fine-tuning.

## 6 Conclusion

This paper presented a unified computer vision pipeline that tracks packages and analyzes their trajectories in real time on edge hardware at a sortation facility. The system integrates a fine-tuned RF-DETR object detector with ByteTrack multi-object tracking, forming a shared backbone from which four task-specific analysis modules derive operational metrics: throughput counting, doubles detection, and reversal detection from the shared trajectories, and dwelling detection from the shared detections.

Operational evaluation demonstrated strong performance across the shared pipeline. On the held-out test set, the fine-tuned RF-DETR Small detector achieved an F1 score of 0.965 and mAP@50 of 0.965. For context, Halawa et al. report F1 values of 0.77–0.87 for RTMDet, YOLO10/12, and RT-DETR variants on a related conveyor dataset; because these results are not from a controlled common-dataset benchmark, they provide context rather than a performance ranking. Downstream, throughput counting achieved 98.05% accuracy across 513 manually counted packages; doubles detection achieved 96.0% precision across 500 reviewed events, including 99.6% precision for reversed doubles, and 66.7% recall on approximately six hours of continuously annotated video; reversal detection achieved 96.6% operational precision across 500 reviewed events and 88.9% recall on the same annotated video; and dwelling detection achieved 100% precision, 96.15% recall, 98.04% F1, and 98.60% accuracy across 500 labeled snapshots. Together, these results quantify the performance of the single shared edge pipeline across detection and all four monitoring outputs.

The principal contribution is a unified backbone for package tracking and trajectory analysis that enables virtual sensors to serve as a potential alternative or complement to physical sensor arrays for selected non-safety-critical monitoring functions while providing richer data. Each detection event carries video evidence, trajectory metadata, and bounding-box geometry that binary sensors cannot capture. This paradigm enables rapid reconfiguration, avoids the signal jittering associated with photoeyes through identity-based event deduplication, and supports the addition of new monitoring tasks without hardware changes.

Future work will pursue four directions: (1) extension to additional facilities to evaluate generalization across diverse conveyor configurations and package distributions, including a camera- and site-held-out evaluation of the detector; (2) integration of reversal and dwelling trend data into predictive maintenance models that anticipate mechanical failures before they cause downtime; (3) exploring model distillation to enable larger RF-DETR variants on current edge hardware, alongside adaptive threshold mechanisms to improve robustness without per-site manual calibration, improved separation of stacked or overlapping packages to raise doubles recall, refinement of the reversal criteria, and an independent evaluation of tracking identity continuity; and (4) integration with programmable logic controllers (PLCs) for proactive interventions, such as canceling a belt reversal when normal single-package flow is detected, and development of additional monitoring tasks such as package damage detection leveraging the same shared tracking backbone.

The current evaluation is limited to a single facility and conveyor type; the architecture's modular design makes it potentially transferable to other operational environments, but this has yet to be demonstrated. More broadly, this work shows that commodity edge hardware combined with modern vision transformers can deliver real-time, high-precision conveyor monitoring in an operational logistics setting, offering a potential alternative or complement to dedicated sensor infrastructure for selected monitoring functions in warehouse automation.

## Data Availability

The datasets presented in this article are not readily available because the dataset used to fine-tune the detection model is proprietary and confidential and cannot be shared. Requests to access the datasets should be directed to Mohammed Mahyoub, mahyoubm@amazon.com.
